# Crystal structures of spinel-type Na_2_MoO_4_ and Na_2_WO_4_ revisited using neutron powder diffraction

**DOI:** 10.1107/S2056989015008774

**Published:** 2015-05-09

**Authors:** A. Dominic Fortes

**Affiliations:** a ISIS Facility, Rutherford Appleton Laboratory, Harwell Science and Innovation Campus, Didcot, Oxfordshire OX11 0QX, England; b Department of Earth Sciences, University College London, Gower Street, London WC1E 6BT, England; c Department of Earth and Planetary Sciences, Birkbeck, University of London, Malet Street, London WC1E 7HX, England

**Keywords:** neutron powder diffraction, sodium molybdate, sodium tungstate

## Abstract

Time-of-flight neutron powder diffraction data have been collected from Na_2_MoO_4_ and Na_2_WO_4_ to a resolution of sin (θ)/λ = 1.25 Å^−1^, which is substanti­ally better than the previous analyses using Mo *K*α X-rays, providing roughly triple the number of measured reflections with respect to the previous studies [Okada *et al.* (1974 ▸). *Acta Cryst.* B**30**, 1872–1873; Bramnik & Ehrenberg (2004 ▸). *Z. Anorg. Allg. Chem.* **630**, 1336–1341]. The unit-cell parameters are in excellent agreement with literature data [Swanson *et al.* (1962 ▸). NBS Monograph No. 25, sect. 1, pp. 46–47] and the structural parameters for the molybdate agree very well with those of Bramnik & Ehrenberg (2004 ▸). However, the tungstate structure refinement of Okada *et al.* (1974 ▸) stands apart as being conspicuously inaccurate, giving significantly longer W—O distances, 1.819 (8) Å, and shorter Na—O distances, 2.378 (8) Å, than are reported here or in other simple tungstates. As such, this work represents an order-of-magnitude improvement in precision for sodium molybdate and an equally substantial improvement in both accuracy *and* precision for sodium tungstate. Both compounds adopt the spinel structure type. The Na^+^ ions have site symmetry .-3*m* and are in octa­hedral coordination while the transition metal atoms have site symmetry -43*m* and are in tetra­hedral coordination.

## Abstract 2

High-precision structural parameters for cubic Na_2_MoO_4_ and Na_2_WO_4_ are reported based on refinement of high-resolution time-of-flight neutron powder diffraction data. Complementary Raman spectra are also provided.

## Chemical context   

Both Na_2_MoO_4_ and Na_2_WO_4_ have rich phase diagrams in pressure and temperature space (Pistorius, 1966 ▸). The stable form at room temperature is the β-Ag_2_MoO_4_ cubic spinel structure type, space group *Fd* 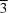 *m*, which has been known for almost a century (Wyckoff, 1922 ▸). Among the alkali metal sulfates, chromates, molybdates and tungstates, only Na_2_MoO_4_ and Na_2_WO_4_ adopt the normal spinel structure at ambient pressure. Li_2_MoO_4_ forms a cubic spinel structure at high pressure (Liebertz & Rooymans, 1967 ▸). Li_2_WO_4_ forms a ‘spinel-like’ phase at high pressure (Pistorius, 1975 ▸; Horiuchi *et al.*, 1979 ▸). Cubic sodium molybdate and sodium tungstate have been examined inter­mittently over subsequent decades using a variety of crystallographic techniques (Lindqvist, 1950 ▸; Becka & Poljak, 1958 ▸; Swanson *et al.*, 1957 ▸, 1962 ▸; Singh Mudher *et al.*, 2005 ▸) and vibrational spectroscopic methods (Busey & Keller, 1964 ▸; Preudhomme & Tarte, 1972 ▸; Breitinger *et al.*, 1981 ▸; Luz Lima *et al.*, 2010 ▸, 2011 ▸), or by nuclear magnetic resonance and quadrupole coupling (Lynch & Segel, 1972 ▸). However, the extant structural information on both phases is derived from X-ray diffraction data of low to modest precision. The first published structure refinement of Na_2_MoO_4_ was only reported recently (Bramnik & Ehrenberg, 2004 ▸) from X-ray powder diffraction data measured to sin (θ)/λ = 0.71 Å^−1^; the last structure refinement of Na_2_WO_4_ was reported by Okada *et al.* (1974 ▸) from X-ray single-crystal diffraction data to sin (θ)/λ = 0.81 Å^−1^. Both compounds are highly soluble in water, crystallizing at room temperature as ortho­rhom­bic dihydrates (space group *Pbca*, Atovmyan & D’yachenko, 1969 ▸; Farrugia, 2007 ▸). Below 283.5 K for the molybdate and 279.2 K for the tungstate, crystals grow with ten water mol­ecules per formula unit (Funk, 1900 ▸; Cadbury, 1955 ▸; Zhilova *et al.*, 2008 ▸). The high solubility in water and propensity towards forming hydrogen-bonded hydrates (unlike the heavier alkali metal molybdates and tungstates) suggests that both compounds would be excellent candidates for formation of hydrogen-bonded complexes with water soluble organics, such as amino acids, producing metal–organic crystals with potentially useful optical properties (*cf*., glycine lithium molybdate; Fleck *et al.*, 2006 ▸).

**Figure 1:**
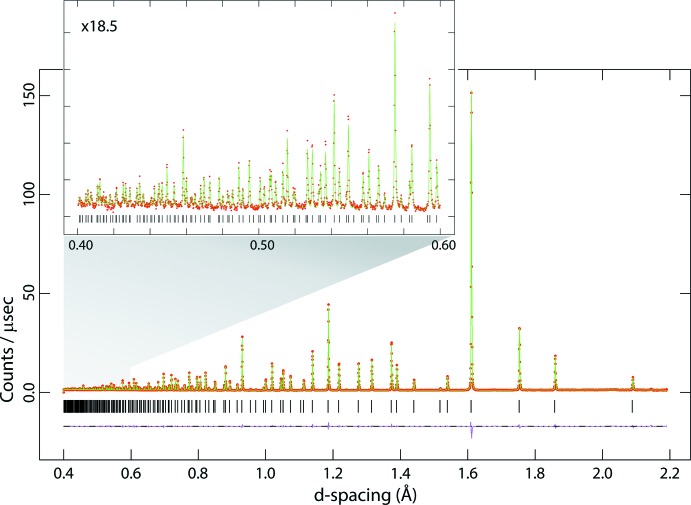
Neutron powder diffraction data for Na_2_MoO_4_; red points are the observations, the green line is the calculated profile and the pink line beneath the diffraction pattern represents Obs − Calc. Vertical black tick marks report the expected positions of the Bragg peaks. The inset shows the data measured at very short flight times (*i.e.*, small *d*-spacing).

In the course of preparing deuterated specimens of the dihydrated and deca­hydrated forms of Na_2_MoO_4_ and Na_2_WO_4_ for neutron diffraction analysis, the anhydrous phases were synthesised and an opportunity arose to acquire neutron powder diffraction data. The advantage of using a neutron radiation probe is that the scattering lengths of the atoms concerned are fairly similar, coherent scattering lengths being 6.715 fm for Mo, 4.86 fm for W, 3.63 fm for Na and 5.803 fm for O (Sears, 2006 ▸). Secondly, with the time-of-flight method, particularly with a very long primary flight path and high-angle backscattering detectors, one can acquire unparalleled resolution at very short flight times (*i.e.*, small *d*-spacings), ensuring an order of magnitude improvement in parameter precision over the previous studies. In this work, usable data were obtained at a resolution of sin (θ)/λ = 1.25 Å^−1^, roughly tripling the number of measured reflections with respect to Okada *et al.* (1974 ▸) and Bramnik & Ehrenberg (2004 ▸). This work provides the most accurate and precise foundation on which to build future discussion of the hydrated forms of Na_2_MoO_4_ and Na_2_WO_4_. Neutron powder diffraction data for Na_2_MoO_4_ and Na_2_WO_4_ are given in Figs. 1 ▸ and 2 ▸.

## Structural commentary   

**Figure 3:**
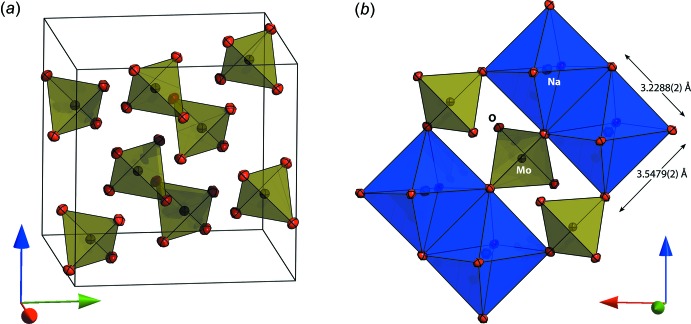
(*a*) Arrangement of molybdate ions in the unit cell of Na_2_MoO_4_; anisotropic displacement ellipsoids are drawn at the 75% probability level. (*b*) Connectivity of the NaO_6_ octa­hedra, with shorter shared edges and longer unshared edges, to the MoO_4_ tetra­hedra in Na_2_MoO_4_; as in (*a*), the ellipsoids are drawn at the 75% probability level.

The structure of both compounds is the normal spinel type with Na^+^ ions on the 16*c* sites in octa­hedral coordination and Mo^6+^/W^6+^ ions on 8*b* sites in tetra­hedral coordination. The coordinating oxygen atoms occupy the 32*e* general positions, their location being defined by a single variable parameter *u*. For ideal cubic close packing, the *u* coordinate adopts a value of 0.25 although for various spinels is found in the range 0.24 to 0.275. In Na_2_MoO_4_ the *u* parameter has a value of 0.262710 (15) and in Na_2_WO_4_ it has a value of 0.262246 (15). The practical consequence of this compared with the ‘ideal’ value of *u* = 0.25 is that the shared edges of the NaO_6_ octa­hedra are shorter than the unshared edges (Fig. 3*b* ▸). In the molybdate, these lengths are 3.2288 (2) and 3.5479 (2) Å, the ratio being 1.0988 (1); in the tungstate, the lengths of the two inequivalent octa­hedral edges are 3.2356 (2) Å and 3.5441 (2) Å, their ratio being 1.0953 (1). The MoO_4_^2−^ and WO_4_^2−^ tetra­hedra have perfect *T_d_* symmetry with Mo—O and W—O bond lengths of 1.7716 (3) and 1.7830 (2) Å, respectively. The unit-cell parameters for both compounds are in excellent agreement with those of Swanson *et al.* (1962 ▸) and the structural parameters for the molybdate agree very well with those of Bramnik & Ehrenberg (2004 ▸). However, the Na_2_WO_4_ structure refinement of Okada *et al.* (1974 ▸) stands apart as being conspicuously inaccurate, giving significantly longer W—O distances, 1.819 (8) Å, and shorter Na—O distances, 2.378 (8) Å, than are reported here or in many other simple tungstates. Indeed the ionic radii of four-coordinated Mo^6+^ and W^6+^ obtained from analysis of a large range of crystal structures are nearly identical, being 0.41 and 0.42 Å, respectively (Shannon, 1976 ▸). The values reported here agree very well with the majority of Mo—O and W—O bond lengths in isolated MoO_4_^2−^ and WO_4_^2−^ tetra­hedral oxyanions from a range of alkali metal and alkaline earth compounds tabulated in the literature (*e.g.*, Zachariasen & Plettinger, 1961 ▸; Gatehouse & Leverett, 1969 ▸; Koster *et al.*, 1969 ▸; Gürmen *et al.*, 1971 ▸; Wandahl & Christensen, 1987 ▸; Farrugia, 2007 ▸; van den Berg & Juffermans, 1982 ▸). As such, this work represents an improvement in accuracy for sodium molybdate and an improvement in both accuracy and precision for sodium tungstate.

## Synthesis and crystallization   

Na_2_MoO_4_·2H_2_O (Sigma Aldrich M1003, > 99.5%) and Na_2_WO_4_·2H_2_O (Sigma Aldrich 14304, > 99.0%) were heated to 673 K in ceramic crucibles for 24 hr. Loss of water was confirmed by Raman spectroscopy; X-ray powder diffraction confirmed the phase identity and purity of the two anhydrous products, Na_2_MoO_4_ and Na_2_WO_4_.

**Figure 4:**
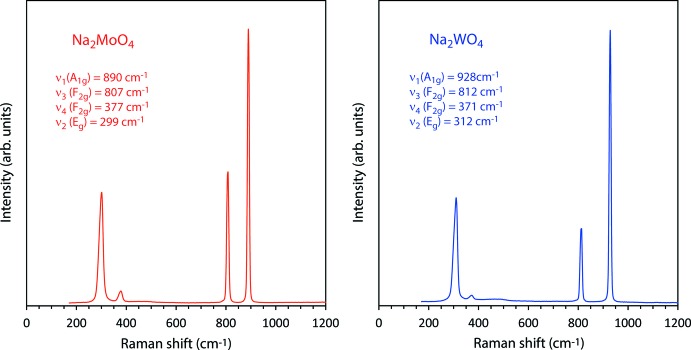
Raman spectra of Na_2_MoO_4_ (left) and Na_2_WO_4_ (right) in the range 0–1200 cm^−1^ (the full range of data to 4000 cm^−1^ is given in the electronic supplement). Band positions and vibrational assignments are indicated. For the tungstate these agree very well with literature values (*e.g.*, Busey & Keller, 1964 ▸) whereas for the molybdate, these data show a systematic shift to lower frequencies by 3–4 wavenumbers with respect to published values (Luz Lima *et al.*, 2010 ▸, 2011 ▸).

Raman spectra were acquired using a B&WTek *i*-Raman plus portable spectrometer; this device uses a 532 nm laser (37 mW power at the fiber-optic probe tip) to stimulate Raman scattering, which is measured in the range 170–4000 cm^−1^ with a spectral resolution of 3 cm^−1^. Data were collected in a series of 20 x 9 sec integrations for Na_2_MoO_4_ and 20 x 7 sec integrations for Na_2_WO_4_; after summation, the background was removed and peaks fitted using Pseudo-Voigt functions in *OriginPro* (OriginLab, Northampton, MA) (Fig. 4 ▸). These data are provided as an electronic supplement in the form of an ASCII file.

## Refinement   

Crystal data, data collection and structure refinement details are summarized in Table 1 ▸. For the neutron scattering experiments, each specimen was loaded into a vanadium tube of 11 mm internal diameter to a depth of approximately 25 mm. The exact sample volume and mass were measured in order to determine the number density for correction of the specimen self-shielding. The samples were mounted on the HRPD beamline (Ibberson, 2009 ▸) at the ISIS neutron spallation source and data were collected in the 10–110 ms time-of-flight window for 2.5 h (Na_2_MoO_4_) and 3.5 h (Na_2_WO_4_). Data were corrected for self-shielding, focussed to a common scattering angle and normalized to the incident spectrum by reference to a V:Nb null-scattering standard before being output in a format suitable for Rietveld refinement with *GSAS/Expgui* (Larsen & Von Dreele, 2000 ▸: Toby, 2001 ▸).

## Supplementary Material

Crystal structure: contains datablock(s) Na~2~MoO~4~, Na~2~WO~4~, New_Global_Publ_Block. DOI: 10.1107/S2056989015008774/wm5152sup1.cif

Rietveld powder data: contains datablock(s) Na2MoO4. DOI: 10.1107/S2056989015008774/wm5152Na2MoO4sup2.rtv

Rietveld powder data: contains datablock(s) Na2WO4. DOI: 10.1107/S2056989015008774/wm5152Na2WO4sup3.rtv

Supporting information file. DOI: 10.1107/S2056989015008774/wm5152sup4.txt

CCDC references: 1063434, 1063433

Additional supporting information:  crystallographic information; 3D view; checkCIF report

## Figures and Tables

**Figure 2:**
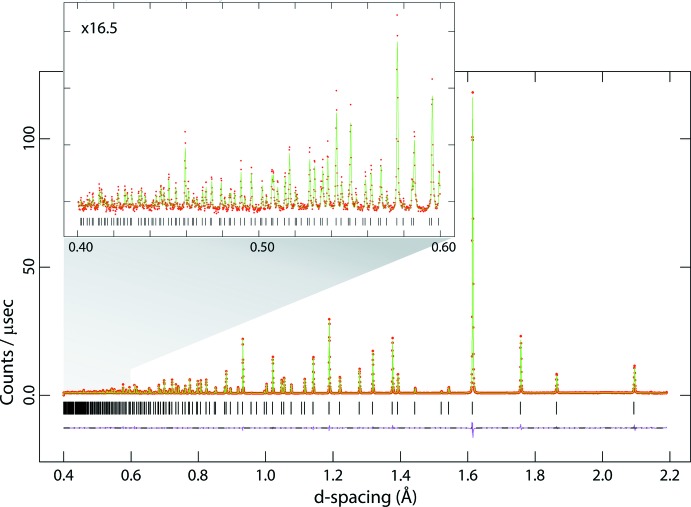
Neutron powder diffraction data for Na_2_WO_4_; red points are the observations, the green line is the calculated profile and the pink line beneath the diffraction pattern represents Obs − Calc. Vertical black tick marks report the expected positions of the Bragg peaks. The inset shows the data measured at very short flight times (*i.e.*, small *d*-spacing).

**Table 1:** Experimental details

	Na_2_MoO_4_	Na_2_WO_4_
Crystal data		
Chemical formula	Na_2_MoO_4_	Na_2_WO_4_
*M*_r_	205.92	293.83
Crystal system, space group	Cubic, *F* *d* 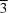 *m*	Cubic, *F* *d* 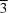 *m*
Temperature (K)	298	298
*a* ()	9.10888(3)	9.12974(4)
*V* (^3^)	755.78(1)	760.98(1)
*Z*	8	8
Radiation type	Neutron	Neutron
(mm^1^)	0.014 + 0.0018 *	0.014 + 0.0097 *
Specimen shape, size (mm)	Cylinder, 25 11	Cylinder, 27 11

Data collection		
Diffractometer	HRPD, high-resolution neutron powder	HRPD, high-resolution neutron powder
Specimen mounting	Vanadium tube	Vanadium tube
Data collection mode	Transmission	Transmission
Scan method	Time of flight	Time of flight
Absorption correction	Analytical	Analytical
2 values ()	2_fixed_ = 168.329	2_fixed_ = 168.329
Distance from source to specimen (mm)	95000	95000
Distance from specimen to detector (mm)	965	965

Refinement		
*R* factors and goodness of fit	*R*_p_ = 0.037, *R*_wp_ = 0.043, *R*_exp_ = 0.022, *R*(*F* ^2^) = 0.06364, ^2^ = 3.842	*R*_p_ = 0.037, *R*_wp_ = 0.044, *R*_exp_ = 0.024, *R*(*F* ^2^) = 0.06245, ^2^ = 3.423
No. of data points	7716	7716
No. of parameters	24	24

Computer programs: *HRPD control software*, *GSAS/Expgui* (Larsen Von Dreele, 2000 ▸; Toby, 2001 ▸), *MANTID* (Arnold *et al.*, 2014 ▸; Mantid, 2013 ▸), *DIAMOND* (Putz Brandenburg, 2006 ▸) and *publCIF* (Westrip, 2010 ▸).

## supplementary crystallographic information

### Crystal data

**Table:**

Na_2_WO_4_	Melting point: 969 K
*M**_r_* = 293.83	Neutron radiation
Cubic, *F**d*3*m*	µ = 0.01+ 0.0097 * λ mm^−^^1^
Hall symbol: -F 4vw 2vw 3	*T* = 298 K
*a* = 9.12974 (4) Å	white
*V* = 760.98 (1) Å^3^	cylinder, 27 × 11 mm
*Z* = 8	Specimen preparation: Prepared at 673 K and 100 kPa
*D*_x_ = 5.129 Mg m^−^^3^	

### Data collection

**Table:**

HRPD, High resolution neutron powder diffractometer	Absorption correction: analytical Data were corrected for self shielding using σ_scatt_ = 28.088 barns and σ_ab_(λ) = 19.361 barns at 1.798 Å during the normalisation procedure. The linear absorption coefficient is wavelength dependent and is calculated as: µ = 0.014 + 0.0097 * λ [mm^-1^]
Radiation source: ISIS Facility, Neutron spallation source	*T*_min_ = 1.000, *T*_max_ = 1.000
Specimen mounting: vanadium tube	2θ_fixed_ = 168.329
Data collection mode: transmission	Distance from source to specimen: 95000 mm
Scan method: time of flight	Distance from specimen to detector: 965 mm

### Refinement

**Table:**

Least-squares matrix: full	Excluded region(s): Data at d-spacings smaller than 0.4 Å were excluded since the counting statistics became progressively poorer at very short flight times due to the lower neutron flux at the shortest wavelengths.
*R*_p_ = 0.037	Profile function: TOF profile function #3 (21 terms). Profile coefficients for exp pseudovoigt convolution [Von Dreele, 1990 (unpublished)] (α) = 0.1603, (β_0_) = 0.026115, (β_1_) = 0.004558, (σ_0_) = 0, (σ_1_) = 237.2, (σ_2_) = 45.0, (γ_0_) = 0, (γ_1_) = 14.21, (γ_2_) = 0, (γ_2s_) = 0, (γ_1e_) = 0, (γ_2e_) = 0, (ε_i_) = 0, (ε_a_) = 0, (ε_A_) = 0, (γ_11_) = 0, (γ_22_) = 0, (γ_33_) = 0, (γ_12_) = 0, (γ_13_) = 0, (γ_23_) = 0. Peak tails ignored where intensity <0.0005x peak. Aniso. broadening axis 0.0 0.0 1.0
*R*_wp_ = 0.044	24 parameters
*R*_exp_ = 0.024	0 restraints
*R*(*F*^2^) = 0.06245	0 constraints
χ^2^ = 3.423	(Δ/σ)_max_ = 0.01
7716 data points	Background function: GSAS Background function # 1 (10 terms). Shifted Chebyshev function of 1st kind 1: 0.884779, 2: 4.212470x10^-2^, 3: 4.210950x10^-2^, 4: -4.489520x10^-2^, 5: -2.683690x10^-2^, 6: -1.892450x10^-2^, 7: -2.248710x10^-2^, 8: -2.821970x10^-3^, 9: 6.467340x10^-3^, 10: 6.167050x10^-3^

### Fractional atomic coordinates and isotropic or equivalent isotropic displacement parameters (Å^2^)

**Table:**

	*x*	*y*	*z*	*U*_iso_*/*U*_eq_	
O	0.262246 (15)	0.262246 (15)	0.262246 (15)	0.01312	
W	0.375	0.375	0.375	0.00903	
Na	0.0	0.0	0.0	0.01538	

### Atomic displacement parameters (Å^2^)

**Table:**

	*U*^11^	*U*^22^	*U*^33^	*U*^12^	*U*^13^	*U*^23^
O	0.01312 (6)	0.01312 (6)	0.01312 (6)	−0.00161 (5)	−0.00161 (5)	−0.00161 (5)
W	0.00903 (11)	0.00903 (11)	0.00903 (11)	0.0	0.0	0.0
Na	0.01538 (11)	0.01538 (11)	0.01538 (11)	−0.00045 (12)	−0.00045 (12)	−0.00045 (12)

### Geometric parameters (Å, º)

**Table:**

W—O	1.7830 (2)	Na^iv^—O^vii^	2.3995 (1)
W—O^i^	1.7830 (2)	Na^iv^—O^viii^	2.3995 (1)
W—O^ii^	1.7830 (2)	Na^iv^—O^ix^	2.3995 (1)
W—O^iii^	1.7830 (2)	Na—Na^iv^	3.2279 (1)
Na^iv^—O	2.3995 (1)	W—Na^iv^	3.7850 (1)
Na^iv^—O^v^	2.3995 (1)	O—O^vi^	3.2356 (2)
Na^iv^—O^vi^	2.3995 (1)	O—O^v^	3.5441 (4)
			
O—W—O^i^	109.4712 (3)	O—Na^iv^—O^v^	95.211 (6)
O—W—O^ii^	109.4712 (3)	O—Na^iv^—O^ix^	180.000 (1)
O—W—O^iii^	109.4712 (3)	O—Na^iv^—O^viii^	84.789 (6)
O^i^—W—O^ii^	109.4712 (3)	O—Na^iv^—O^vii^	95.211 (6)
O^i^—W—O^iii^	109.4712 (3)	O^v^—Na^iv^—O^vi^	180.000 (1)
O^ii^—W—O^iii^	109.4712 (3)	W—O—Na^iv^	129.043 (4)
O—Na^iv^—O^vi^	84.789 (6)		

Symmetry codes: (i) −*x*+3/4, *y*, −*z*+3/4; (ii) −*x*+3/4, −*y*+3/4, *z*; (iii) *x*, −*y*+3/4, −*z*+3/4; (iv) −*x*+1/4, −*y*+1/4, *z*; (v) *x*, −*y*+1/4, −*z*+1/4; (vi) −*y*+1/2, *x*+1/4, *z*−1/4; (vii) −*x*+1/4, *y*, −*z*+1/4; (viii) *y*+1/4, −*x*+1/2, *z*−1/4; (ix) −*y*+1/2, −*x*+1/2, −*z*.
